# Psychiatric presentation of patients with acute SARS-CoV-2 infection: a retrospective review of 50 consecutive patients seen by a consultation-liaison psychiatry team

**DOI:** 10.1192/bjo.2020.85

**Published:** 2020-09-10

**Authors:** Yousaf Iqbal, Majid Ali Al Abdulla, Sultan Albrahim, Javed Latoo, Rajeev Kumar, Peter M. Haddad

**Affiliations:** Psychiatric Hospital, Hamad Medical Corporation, Qatar; Psychiatric Hospital, Hamad Medical Corporation; and College of Medicine, Qatar University, Qatar; Psychiatric Hospital, Hamad Medical Corporation, Qatar; Psychiatric Hospital, Hamad Medical Corporation, Qatar; Psychiatric Hospital, Hamad Medical Corporation, Qatar; Psychiatric Hospital, Hamad Medical Corporation, Qatar; and College of Medicine, Qatar

**Keywords:** COVID-19, delirium, consultation-liaison psychiatry, psychosis, aetiology

## Abstract

**Background:**

Reports of psychiatric morbidity associated with severe acute respiratory syndrome coronavirus 2 (SARS-CoV-2) infection tend to be limited by geography and patients’ clinical status. Representative samples are needed to inform service planning and research.

**Aims:**

To describe the psychiatric morbidity associated with SARS-CoV-2 infection (confirmed by real-time polymerase chain reaction) in referrals to a consultation-liaison psychiatry service in Qatar.

**Method:**

Retrospective review of 50 consecutive referrals.

**Results:**

Most patients were male. Median age was 39.5 years. Thirty-one patients were symptomatic (upper respiratory tract symptoms or pneumonia) for coronavirus disease 2019 (COVID-19) and 19 were asymptomatic (no characteristic physical symptoms of COVID-19 infection). Seventeen patients (34%) had a past psychiatric history including eight with bipolar I disorder or psychosis, all of whom relapsed. Thirty patients (60%) had physical comorbidity. The principal psychiatric diagnoses made by the consultation-liaison team were delirium (*n* = 13), psychosis (*n* = 9), acute stress reaction (*n* = 8), anxiety disorder (*n* = 8), depression (*n* = 8) and mania (*n* = 8). Delirium was confined to the COVID-19 symptomatic group (the exception being one asymptomatic patient with concurrent physical illness). The other psychiatric diagnoses spanned the symptomatic and asymptomatic patients with COVID. One patient with COVID-19 pneumonia experienced an ischaemic stroke. Approximately half the patients with mania and psychosis had no past psychiatric history. Three patients self-harmed. The commonest psychiatric symptoms were sleep disturbance (70%), anxiety (64%), agitation (50%), depressed mood (42%) and irritability (36%).

**Conclusions:**

A wide range of psychiatric morbidity is associated with SARS-CoV-2 infection and is seen in symptomatic and asymptomatic individuals. Cases of psychosis and mania represented relapses in people with schizophrenia and bipolar disorder and also new onset cases.

## Background

Coronavirus disease 2019 (COVID-19) is caused by infection with severe acute respiratory syndrome coronavirus 2 (SARS-CoV-2) and was first reported in Wuhan, Hubei Province, China, in December 2019.^1^ The disease spread rapidly across the world and was declared a pandemic by the World Health Organization in March 2020.^2^ As of 28 June 2020 there had been over 10 million cases reported worldwide and over 500 000 deaths.^3^ A significant proportion of those testing positive for SARS-CoV-2 are asymptomatic; estimates range from 5 to 80%.^4^ At a community level, most symptomatic individuals are mildly ill, but a minority develop severe complications including pneumonia, acute respiratory distress syndrome (ARDS), sepsis and organ failure. The case fatality rate varies markedly between countries, but worldwide is approximately 5%.^3^ To limit the spread of infection many countries have introduced social restrictions (‘lockdowns’) although some countries are now seeing these being cautiously lifted.

## COVID-19 and psychiatric morbidity

High rates of psychiatric morbidity have occurred in association with previous epidemics of respiratory viruses including severe acute respiratory syndrome (SARS), Middle East respiratory syndrome (MERS), and H1N1 influenza.^5,6^ The SARS-CoV-2 pandemic is only a few months old but there are increasing reports of psychiatric morbidity.^5,7^ This can arise through psychosocial stressors irrespective of infection, for example fear, isolation, bereavement, unemployment and financial difficulties. A positive test for SARS-CoV-2 may lead to additional stress, for example that associated with quarantine.^8^ COVID-19 infection, its complications and its treatment can have direct effects on the brain leading to neuropsychiatric disorders including seizures, delirium, demyelination, encephalopathies and stroke.^5,9^

Current studies on psychiatric presentations of SARS-CoV-2 have several limitations. In terms of geography, most are from Wuhan, China.^5^ A broader range of data is required as patterns of psychiatric morbidity may vary between countries reflecting differences in lockdown measures, infection and mortality rates, sociodemographic and cultural factors, and access to physical and mental healthcare. Another limitation is that some studies are restricted to assessing one aspect of psychopathology, for example anxiety and depression^7^ or neuropsychiatry.^5,9^ Other studies focus on a narrow patient group, for example those who died^10,11^ or were admitted to intensive care units.^12^ Finally, all current studies in hospital settings have restricted themselves to symptomatic patients with COVID-19, although psychiatric consultation-liaison services will also be referred patients who have tested positive for SARS-CoV-2 but are physically asymptomatic.

## Aims

The current study aimed to complement existing data by characterising the psychiatric morbidity associated with acute SARS-CoV-2 infection in patients referred to a consultation-liaison psychiatry service in Qatar. As such it offers a broad clinical picture of the psychiatric problems associated with acute SARS-CoV-2 infection, occurring in a general hospital setting, and including patients who are symptomatic and asymptomatic for COVID-19 infection. These data should assist with service planning and future research.

The first case of COVID-19 in Qatar was reported on 27 February 2020.^13^ The following month the government introduced a series of lockdown measures including the closure of schools and universities, non-essential shops, parks and public beaches. At the time of writing (1 July 2020) Qatar has the highest total number of confirmed cases per capita of any country in the world at 31 116 cases per million population.^3^ This reflects high rates of testing and contact tracing and the high-density accommodation where most low-income migrant workers live that facilitates the spread of infection. However, the case mortality rate is one of the lowest in the world.^3^ This may reflect the high quality of Qatar's healthcare system and the high proportion of the population who are young.

## Method

### Hamad Medical Corporation consultation-liaison service

Three hospitals covered by the consultation-liaison team are included in this study, namely Hazm Mebaireek General Hospital, the Communicable Disease Centre Hospital and Hamad General Hospital. All are situated in Doha, the capital of Qatar. They are run by Hamad Medical Corporation (HMC), Qatar's primary government funded healthcare service that provides free hospital care to all Qatari residents, irrespective of nationality. The first two were the only two designated hospitals for managing COVID-19 patients at the time the data was collected. Across all three hospitals, consultation-liaison teams receive referrals electronically from doctors in other specialties. A psychiatrist triages the referrals into emergency and urgent cases. All patients classified as emergency cases are assessed on the same day within 4 h, and all those classified as urgent cases are evaluated within 24 h. All psychiatric assessments are completed by consultant psychiatrists or board-qualified psychiatry fellows under the supervision of a consultant psychiatrist. Patients are assessed face to face and/or virtually through a video/phone call. Doha is a highly multicultural city; where possible, psychiatrists who speak the same language as the patient are allocated to conduct assessments; otherwise, translators are used.

### Design, approval and date extraction

The study design was a retrospective case-note review. The study received approval from the HMC Institutional Review Board (IRB) (MRC-05–072). Individual patient consent was not deemed necessary by the IRB. Registers kept by the consultation-liaison teams at each hospital were reviewed to identify the first 50 consecutive referrals who met the following three inclusion criteria:

- age 18 years and above;
- a positive antigen test for SARS-CoV-2 (real-time polymerase chain reaction test) taken during the patient's current period of hospital admission;
- referral from a hospital ward or the emergency department.

A proforma was used to record relevant information from the electronic medical records. Data was extracted by two members of the research team (Y.I. and S.A.) who held regular discussions to ensure consistency in data recording. Any rating uncertainties were resolved by discussion with a third team member. The proforma collected demographic characteristics and details of physical comorbidities, past psychiatric history, severity of COVID-19 infection and COVID-19 treatment during the current hospital episode. Details of the consultation-liaison assessment that were recorded included psychiatric symptoms/signs and clinical diagnosis as recorded in the notes. Diagnoses reflected clinical judgement and were not operationally defined. Data was extracted for the whole of the current hospital episode; in some cases, the consultation-liaison team conducted several assessments during this period. The severity of COVID-19 infection related to the most severe illness during the current hospital admission episode and was made by the medical team (not the consultation-liaison team) using a five-point system used in HMC hospitals (Communicable Disease Centre Criteria as per HMC treatment protocol) as follows:

- asymptomatic: i.e. no characteristic physical symptoms of COVID-19 infection;
- mild COVID-19: uncomplicated upper respiratory tract viral infection, may have non-specific symptoms such as fever, cough, sore throat, nasal congestion, malaise, headache, muscle pain or malaise. Elderly people and individuals who are immunosuppressed may present with atypical symptoms;
- mild pneumonia: patient with pneumonia and no signs of severe pneumonia;
- severe pneumonia: fever or suspected respiratory infection, plus one of: (i) respiratory rate >30 breaths/min, (ii) severe respiratory distress, or (iii) SpO_2_ <90% on room air;
- critical disease: acute respiratory distress syndrome, sepsis, septic shock.

Analysis was largely by descriptive statistics.

## Results

Table 1 shows the sociodemographic and clinical details of the 50 patients. In terms of COVID-19 illness severity, 19 patients were asymptomatic, 8 patients had mild COVID-19 (i.e. symptoms of upper respiratory tract infection alone) and 23 patients had pneumonia of varying severity, with 7 of these being critically ill. Two of the 50 patients died from COVID-19. A total of 17 patients had a past psychiatric history including 8 who had psychosis or bipolar I disorder, all of whom presented with a relapse in the study period.

**Table 1:** Sociodemographic and medical details of 50 consecutive patients positive for severe acute respiratory syndrome coronavirus 2 (SARS-CoV-2) referred to Hamad Medical Corporation consultation-liaison psychiatry service

Characteristic	Value
Age, years	
Median	39.5
Mean	43.9
Range	18–86
Men, *n* (%)	48 (96)
Nationality, *n* (%)	
South Asian (Indian, Pakistani, Bangladeshi, Nepalese, Afghan)	33 (66)
Qatari	9 (18)
Other Arab	3 (6)
Philippines	2 (4)
White	2 (4)
African	1 (2)
Source of psychiatric referral, *n* (%)	
Ward referral	40 (80)
Emergency department	10 (20)
Physical comorbidity present: includes: cardiovascular disease, diabetes, liver disease, lung disease, cancer, chronic kidney disease	30 (60)
Past psychiatric history, *n* (%)	
None	33 (66)
Bipolar I disorder	5 (10)
Depression, anxiety disorder	5 (10)
Schizophrenia, psychosis	3 (6)
Dementia	3 (6)
Alcohol dependence	1 (2)
Severity of COVID infection (criteria),^a^ *n* (%)	
Asymptomatic	19 (38)
Mild COVID	8 (16)
Mild COVID pneumonia	8 (16)
Severe COVID pneumonia	8 (16)
Critical	7 (14)
Details of COVID treatment, *n* (%)	
Supplementary oxygen	20 (40)
Steroids	13 (26)
Antibiotics/ antivirals	36 (72)
Tocilizumab	7 (14)
Psychiatry diagnosis^b^ made by consultation-liaison team, *n* (%)	
Delirium	13 (26)
Non-affective psychosis	9 (18)
Acute stress reaction	8 (16)
Anxiety disorders	8 (16)
Depression (includes 1 patient with depressive psychosis)	8 (16)
Mania	8 (16)
Dementia (all cases present pre-COVID-19)	3 (6)
Metabolic encephalopathy	1 (2)
Herpes simplex encephalitis	1 (2)
Alcohol withdrawal	1 (2)
No psychiatric diagnosis	1 (2)
Self-harm	3 (6)
Psychiatric outcome on discharge, *n* (%)	
Resolved at time of hospital discharge	15 (30)
Improved but not fully resolved	31 (62)
Continued or worsened (2 patients died with COVID-19)	2 (4)

a. See main text for definitions of severity of COVID-19 using the Communicable Disease Centre Criteria as per Hamad Medical Corporation treatment protocol.

b. The sum of psychiatry diagnoses exceeds >50 as some patients had more than one psychiatric diagnosis. These comprise patients with delirium superimposed on pre-existing dementia (*n* = 3), delirium associated with metabolic encephalopathy (*n* = 1) and herpes simplex encephalitis/alcohol withdrawal (*n* = 1), delirium followed by depression (*n* = 1), anxiety and depression in the same patient (*n* = 4).

A psychiatric diagnosis was made by the consultation-liaison team in 49 of the 50 patients. The psychiatric diagnoses included delirium (*n* = 13), non-affective psychosis (*n* = 9), acute stress reaction (*n* = 8), anxiety disorder (*n* = 8), mania (*n* = 8) and depression (*n* = 8). All these psychiatric diagnoses were seen in both the COVID-19 symptomatic and asymptomatic patients. However, all but one case of delirium occurred in the symptomatic COVID-19 group. The one case of delirium in an asymptomatic patient with COVID-19 appeared to be the result of alcohol withdrawal and herpes simplex encephalitis. Among the 12 cases of delirium associated with the COVID-19 symptomatic group was one patient with a metabolic encephalopathy and three patients with pre-existing dementia.

Approximately half of the patients with a current diagnosis of mania (3/8) and a current non-affective psychosis (5/9) had no past psychiatric history i.e. their first episode of illness coincided with their being positive for SARS-CoV-2. There were three cases of non-fatal self-harm (one overdose, one self-laceration, one jumping from a height). Two of these individuals had depression and the third had an acute and transient psychosis. In each case the psychiatric disorder appeared reactive to the psychosocial impact of the pandemic. Of these three individuals, one had a past psychiatric history and two were asymptomatic for COVID-19 and the third had mild disease. An additional patient with mania ingested antiseptic, not to harm himself but to reduce his risk of becoming ill with COVID-19. He reported doing this after hearing a media report of the President of the USA advocating disinfectants as a treatment for COVID-19.^14^

In total, 22 (44%) of the patients underwent brain imaging during the current hospital admission. One patient developed COVID-related ARDS and soon after a new onset of left-sided weakness. A diagnosis of stoke was made. Magnetic resonance imaging (MRI) showed multifocal bilateral acute infarcts more prominent on the right side, an occluded right internal carotid artery and minimal microhaemorrhages. He was referred to the consultation-liaison team for advice on the management of behavioural disturbance associated with delirium. A second patient with critical severity COVID-19 had MRI changes suggestive of small subcortical infarcts.

Table 2 records the prevalence of psychiatric symptoms recorded during the consultation-liaison assessment. The commonest symptoms recorded in the notes were sleep disturbance (*n* = 35, 70%), anxiety (*n* = 32, 64%), agitation (*n* = 25, 50%), depressed mood (*n* = 21, 42%) and irritability (*n* = 18, 36%).

**Table 2:** Prevalence of psychiatric signs and symptoms reported by consecutive patients with severe acute respiratory syndrome coronavirus 2 (*n* = 50) referred to the psychiatric consultation-liaison service (only symptoms reported by ten or more patients are reported)

Symptom	Prevalence of symptom, *n* (%)
Insomnia	35 (70)
Anxiety	32 (64)
Agitation	25 (50)
Depressed mood	21 (42)
Irritability	18 (36)
Change in appetite	17 (34)
Disorientation and/or confusion	15 (30)
Aggression	13 (26)
Delusions	12 (24)
Euphoria/elation	11 (22)
Thoughts of self-harm	10 (20)
Impaired concentration	10 (20)
Impaired memory	10 (20)
Fatigue	7 (14)
Visual hallucinations	6 (12)
Disinhibition	5 (10)

## Discussion

### Strengths and weaknesses

Strengths of the current study are that consecutive patients meeting inclusion criteria were included and recruitment was from three hospitals in Qatar, two of which were COVID-19 designated hospitals. We included symptomatic and asymptomatic patients who had tested positive for SARS-CoV-2 during their current hospital admission in order to provide a comprehensive picture of psychiatric morbidity associated with acute infection. Data was extracted by two members of the research team who consulted regularly to enhance consistency of ratings. The main weakness is that this is a retrospective case-note review and the data is dependent on the quality of the medical notes. As we examined referrals to a consultation-liaison service, our data may underestimate neuropsychiatric cases that lie more to the neurology end of the spectrum as these may have been referred to neurology rather than to psychiatry.

### Patient age

The median age in our sample was 39.5 years and all but two patients were male. This is consistent with the demographics of Qatar where a high proportion of the population are migrant male labourers from South Asia^15^ and the median age of the population is 32 years.^16^ However, individuals from a wide range of nationalities were seen. Our median age contrasts with that of 71 years reported in a recent UK series of patients with neurological and neuropsychiatric complications of COVID-19.^9^ The mean age of our sample (43.9 years) is also lower than that reported in each of ten publications/pre-prints in a review of acute psychiatric and neuropsychiatric outcomes of SARS-CoV-2 infections.^5^

### Asymptomatic patients

The high proportion of patients who were asymptomatic for COVID-19 (38%) in our series reflects several factors. Some were patients who were transferred to hospital from a quarantine centre after they developed psychiatric symptoms, some presented directly to hospital with psychiatric symptoms and medical screening revealed them to be positive for SARS-CoV-2 and some people who screened positive for SARS-CoV-2 during the early stage of the pandemic were admitted to hospital even though asymptomatic in terms of COVID-19 symptoms.

### Interpretation of our findings

Previous commentators have drawn attention to the concern that the pandemic may have a disproportionate impact on those with pre-existing psychiatric problems.^17,18^ Community studies are needed to examine this. However, the fact that a third of our sample had a past psychiatric history, and of these nearly half had a psychotic disorder or bipolar I disorder, suggests that those with prior mental health problems are particularly vulnerable to develop further mental health problem during the pandemic. Conversely, we also observed individuals who developed psychosis and mania for this first time in association with SARS-CoV-2 infection. Further research is needed to clarify to what extent the mechanism in such cases is psychosocial and/or organic.

Despite the relatively young mean age of the sample, the most frequent psychiatric diagnosis was delirium. This may partly reflect the high rate of physical comorbidity (60%) in the sample. One patient with delirium had mild COVID-19 (i.e. no symptoms of pneumonia) and no other evidence of physical illness (this included normal brain MRI). This raises the possibility that the delirium could have been the result of a direct neurotoxic effect of SARS-CoV-2, another mechanism related to the virus or a cause unrelated to infection that was not identified during the admission.

Our series included one patient who had a stroke, due to occlusion of his right internal carotid artery, soon after developing COVID-19 pneumonia. Large vessel occlusion and ischaemic strokes have been reported in association with COVID-19.^19^ In a UK study, 82% of patients with SARS-CoV-2 infections who presented with a cerebrovascular event (mostly infarcts) were over 60 years old.^9^ Infarcts may reflect proinflammatory cytokines leading to a prothrombotic state and/or increased antiphospholipid antibodies. We also observed a 41-year-old previously healthy patient who developed small subcortical ischaemic changes in association with critical COVID-19.

### Social media and COVID-19

Excessive use of social media to access COVID-19 related information has been associated with anxiety.^20^ Within our case series, there were several individuals in whom media reports appeared to have had a negative impact on their mental state and behaviour. Conversely, the media can be beneficial in keeping people informed during the pandemic and social media can help combat isolation allowing people to remain in contact with family and friends during lockdown and quarantine.

### Most prevalent psychiatric symptoms

The most prevalent psychiatric symptoms were insomnia (70%), anxiety (64%), agitation (50%), depressed mood (42%) and irritability (36%). This pattern is similar to that seen with previous acute coronavirus epidemics.^5^ A combined analysis of two studies that assessed psychiatric symptoms in acute SARS and MERS infection with a symptom checklist found insomnia and anxiety to be the most prevalent symptoms with depressed mood appearing in fifth place.^5^

The 50 cases we report were referred during the early part of the pandemic in Qatar. Treatment for COVID-19 will almost certainly improve as new treatments are found to be effective. For example, in mid-June preliminary data showed that dexamethasone reduced mortality in patients with COVID-19 who required oxygen or ventilator support.^21^ Improved treatment may have an impact on associated psychiatric morbidity by reducing health anxiety and the severity of COVID-19 illness.

### Directions for future studies

This study focused on psychiatric presentations associated with acute infection. Future studies will be necessary to determine the pattern of psychiatric morbidity months and years after acute infection. High rates of depression,^22^ anxiety^23^ and post-traumatic stress disorder^24^ are seen in survivors of critical illnesses 1 year later. Some cases may represent chronic disorders that started during or soon after an acute illness whereas others may represent illnesses commencing after a latent period. It is possible that SARS-CoV-2 may also lead to latent neuropsychiatric disorders. This is partly based on the 1918 flu pandemic being suggested as a cause of encephalitis lethargica and subsequent post-encephalitic Parkinsonism.^25^ Prospective studies using diagnostic instruments are needed to more accurately characterise the psychiatric morbidity associated with SARS-CoV-2, both during and after infection.

### Implications

In conclusion, a wide range of psychiatric morbidity was seen in association with SARS-CoV-2 infection and occurred in those who were asymptomatic and symptomatic for COVID-19 infection. The most common syndrome in this study was delirium, despite the relatively young age of the patients. Consultation-liaison services in countries with older populations may encounter an even higher proportion of cases of delirium. They may also expect to see more cases of stroke associated with COVID-19. Another factor that may have impacted on our results is that a high proportion of the expatriate Qatar workforce live away from their families, who remain in their home countries. This may limit psychosocial support at times of stress and predispose to functional psychiatric illness.

Consultation-liaison services have a crucial contribution to make to patient care during the pandemic. The high prevalence of neuropsychiatric disorders highlights the needs for such services to work closely with medical services. Consultation-liaison services also need to work closely with hospital- and community-based psychiatric services to ensure effective follow-up for patients after discharge from the general hospital. Increased community support for those with existing mental illness may reduce the impact of the pandemic in this group and serve to prevent some of the morbidity noted in this study. We hope that the data we report will assist with planning consultation-liaison services and increase understanding of the psychiatric morbidity associated with the pandemic.

## Data Availability

The data that support the findings of this study are available from the corresponding author upon reasonable request and pending additional ethical approval.

## Supplementary material

For supplementary material accompanying this paper visit http://dx.doi.org/10.1192/bjo.2020.85. click here to view supplementary material
